# Bacteria drug resistance profile affects knee and hip periprosthetic joint infection outcome with debridement, antibiotics and implant retention

**DOI:** 10.1186/s12891-020-03570-1

**Published:** 2020-08-24

**Authors:** Bruno Alves Rudelli, Pedro Nogueira Giglio, Vladimir Cordeiro de Carvalho, José Ricardo Pécora, Henrique Melo Campos Gurgel, Ricardo Gomes Gobbi, José Riccardo Negreiros Vicente, Ana Lucia Lei Munhoz Lima, Camilo Partezani Helito

**Affiliations:** Instituto de Ortopedia e Traumatologia do Hospital das Clínicas da Faculdade de Medicina da Universidade de São Paulo, 333 Dr Ovídeo Pires de Campos St, São Paulo, Zip Code 05403-010 Brazil

**Keywords:** Periprosthetic joint infection, Total hip arthroplasty, Total knee arthroplasty, Multidrug resistant bacteria, Complications, Acute post-operative infection, DAIR (debridement, Antibiotics and implant retention)

## Abstract

**Background:**

Evaluate the effect of bacteria drug resistance profile on the success rates of debridement, antibiotics and implant retention.

**Methods:**

All early acute periprosthetic infections in hip and knee arthroplasties treated with DAIR at our institution over the period from 2011 to 2015 were retrospectively analyzed. The success rate was evaluated according to the type of organism identified in culture: multidrug-sensitive (MSB), methicillin-resistant *Staphylococcus aureus* (MRSA), multidrug-resistant Gram-negative bacteria (MRB) and according to other risk factors for treatment failure. The data were analyzed using univariate and multivariate statistics.

**Results:**

Fifty-seven patients were analyzed; there were 37 in the multidrug-sensitive bacteria (MSB) group, 11 in the methicillin-resistant *Staphylococcus aureus* (MRSA) group and 9 in the other multidrug-resistant Gram-negative bacteria (MRB) group. There was a statistically significant difference (*p* < 0.05) in the treatment failure rate among the three groups: 8.3% for the MSB group, 18.2% for the MRSA group and 55.6% for the MRB group (*p* = 0.005). Among the other risk factors for treatment failure, the presence of inflammatory arthritis presented a failure rate of 45.1 (*p* < 0.05).

**Conclusion:**

DAIR showed a good success rate in cases of early acute infection by multidrug-sensitive bacteria. In the presence of infection by multidrug-resistant bacteria or association with rheumatic diseases the treatment failure rate was higher and other surgical options should be considered in this specific population. The MRSA group showed intermediate results between MSB and MRB and should be carefully evaluated.

## Background

Periprosthetic joint infection (PJI) is one of the most demanding and challenging complications for orthopedic surgeons [1]. It has a low incidence in total hip (THA) and total knee (TKA) arthroplasty, ranging between 0.2 and 2% of primary procedures [2, 3]. Despite its low incidence, PJI is the third leading cause of revision THA in the United States, representing 14.8% of all revision surgeries [4]. The ageing population has led to an increase number of arthroplasties performed worldwide, and the amount of cases of PJI is estimated to grow significantly in the coming decades [3]. Kurtz et al. [5] estimated that by the year 2030, there will be a 613% increase in the demand for TKA and a 174% increase for THA as a result of degenerative processes.

The most appropriate treatment for PJI is still debated. Among the many options for treatment; Debridement, Antibiotics and Implant Retention is most indicated in cases of early acute an hematogenic acute infections. It has the advance of being little aggressive while maintaining the implants, though its success rate is debatable in the literature [6, 7].

In acute cases where a mature biofilm has not yet been formed on the surface of the prosthetic material, the most used treatment is DAIR with exchange of the modular components [6, 8]. The factors that contribute to poor prognosis in the treatment of PJI include age, gender, ethnicity, diabetes, rheumatic disease, obesity, malnutrition and multidrug-resistant infections [2, 9–11].

The emergence of multidrug-resistant bacteria in hospital environments is a phenomenon that accompanies the development of new antimicrobials and their indiscriminate use. Some studies have demonstrated that infections by these agents represent an important risk factor for PJI treatment failure [2]. The universe of drug resistant bacteria is very complex with a miscellaneous of pathogens with resistance profiles ranging to one or few commonly used antibiotics, in particular methicillin-resistant *Staphylococcus aureus* (MRSA) and vancomycin-resistant *Enterococcus spp* (VRE), to multi-drug resistant bacteria, such as multidrug-resistant *Pseudomonas* and *Acinetobacter* [2, 11, 12]. It is important to understand the difference between such organisms, as the success rates deteriorate with the increase of drug resistance profile [2].

Some authors [12] have suggested that infections in TKA by multidrug-resistant microorganisms other than MRSA have worse results in the two-stage exchange of modular components, with an increase incidence of prosthesis failure and even an increase in limb amputation. However, to the best of our knowledge there are no studies that compare the success rate of DAIR for acute infections caused by resistant Gram-negative microorganisms or other microorganisms.

The objective of the present study is to analyze the influence of the infecting organism in the success rate of treatment in acute periprosthetic knee and hip infections treated with DAIR. Our hypothesis is that both MRSA infections and infections by multidrug-resistant Gram-negative bacteria will have poorer results than infections caused by multi-sensitive bacteria in the matter of prosthesis retention.

## Methods

A retrospective study was carried out using data collected prospectively for all acute PJI of total knee and hip arthroplasty from our institution over the period from 2011 to 2015. We included all cases of acute infection (up to 3 weeks after the index surgery) that meet the criteria of PJI defined by the Musculoskeletal Infection Society [13] (Table 1) and had identification of the infecting organism in cultures from the initial diagnostic aspiration or intraoperative samples.

**Table 1:** Criteria for PJI

Based on the proposed criteria, PJI is defined as follows	
(1) Presence of fistula communicating to the prosthesis; or	
(2) The same microorganism isolated by culture in at least two separate tissue or fluid samples obtained from the affected joint or prosthesis; or	
(3) Four of the following six criteria:	
(a) Elevation of erythrocyte sedimentation rate (ESR) and serum C-reactive protein (CRP) concentration,	
(b) Elevation of the leukocyte count in the synovial fluid,	
(c) Elevation of the percentage of neutrophils,	
(d) Presence of purulence in the affected joint,	
(e) Isolation of only one microorganism in a culture of the tissue or periprosthetic fluid, or	
(f) More than five neutrophils per high power field in at least five high power fields observed from the histological analysis of the periprosthetic tissue at 9400X magnification.	

Cases of partial arthroplasty, sub-acute or chronic infections (more than 3 weeks after arthroplasty), cases with negative culture results or previous infections in the affected joint were not included in this study. Cases where the treatment used was not DAIR were also excluded from the analysis.

All specimens collected for culture were sent to the microbiology laboratory in sterile containers with thioglycolate broth, and the joint fluid samples collected by aspiration were sent for analysis in blood culture bottles.

The following data were collected and evaluated: gender, age, joint affected, comorbidities (smoking, diabetes mellitus and inflammatory diseases), time of post-arthroplasty infection, microorganism responsible for the infection and its sensitivity profile and the success or failure of the initial treatment. Success was defined as implant retention without the need for new surgical procedures after the first DAIR procedure.

Patients were divided into three groups according to the bacteria that caused the infection: multidrug-sensitive bacteria (Group 1), methicillin*-*resistant *Staphylococcus aureus* (MRSA) (Group 2) and Gram negative multidrug-resistant bacteria (Group 3), as to compare success rates among them.

All patients received antibiotic prophylaxis 1 h previously to skin incision. Teicoplanin (400 mg) and amikacin (500 mg) were the antibiotics of choice when pre-operative culture was not available, which occurred in 52 of the 57 cases (between 2011 and 2015 it was not a routine in our institution to perform pre-operative aspiration for all cases of suspected PJI). In the 5 cases that had a positive preoperative culture obtained by joint aspiration, the antibiotics were target to the specific antibiotic sensitivity profile of the pathogen. None of these 5 cases were multi-drug resistant bacteria. After surgery patients received at least 2 weeks of intravenous antibiotic (teicoplanin 400 mg 12/12 h in the first 48 h, then 400 mg once a day – for gram positive bacteria – and amikacin 500 mg once a day – for gram negative bacteria) followed by 4 weeks of oral antibiotics, if it was possible based on bacteria resistance profile.

Extensive debridement was performed always in the presence of the assistant surgeon supervision. The irrigation was carried out with at least 10 l of sodium chloride 0,9% solution, no local antimicrobial agents were utilized during or after irrigation. The modular components were exchanged (polyethylene liner in total knee arthroplasty and polyethylene liner and femoral head in total hip arthroplasty) and the implant fixation stability were tested. After the debridement an irrigation, and prior to implantation of the new liner and femoral head, the surgical team exchanged gloves and re-draping was performed. At least five samples among periprosthetic tissue, bone and joint fluid were collected during the procedure and prior to debridement and irrigation. In all cases we used drains that were removed 24 to 48 h after surgery depending upon the drain out. The mean surgical time was 75 min (ranging between 50 to 90 min).

Normally distributed variables were described as mean ± standard deviation, and not normally distributed ones as median (interquartile range). For univariate analyses, the Kruskal-Wallis test for comparison of independent samples was used for continuous variables, and Fisher’s exact test was used for categorical variables. To evaluate the independent determinants of the outcome ‘treatment success or failure’, multivariate logistic regression analysis was used. *P* < 0.05 was considered statistically significant. The sample size was not calculated since all available patients were included in the study. The statistical software SPSS 22 (IBM Corp.) was used for the analyses. Multivariate logistic regression was performed to investigate the effects of gender, joint affected, diabetes, smoking, inflammatory disease and type of bacteria in the outcome ‘treatment success’ (Table 2). The model was statistically significant, with χ2 = 024.938 and *p* < 0.001, explaining 67.7% (Nagelkerke R^2^) of the treatment success variance, and correctly classified 89.5% of the cases.

**Table 2:** Multivariate analysis of treatment failure predictors *,**

	*P* value	Adjusted risk ratio	95% confidence interval
gender	0.123	0.103	0.006–1.84
age	0.782	0.967	0.766–1.22
time of post-arthroplasty infection (days)	0.108	1.19	0.962–1.47
joint (knee or hip)	0.753	1.461	0.137–15.52
diabetes	0.905	1.248 45.101	0.033–15.52
**rheumatic disease**	**0.04**	**45.101**	**1.18–1723.14c2.641–1296.90 1.039–2402**
multidrug-sensitive bacteria	0.034	–	–
**methicillin-resistant Staphylococcus aureus**	**0.01**	**58.527**	**2.641–1296.90**
other multidrug-resistant bacteria	0.048	49.967	1.039–2402
constant	0.999	0	–

*Multivariate logistic regression was performed to investigate the effects of gender, joint affected, diabetes, smoking, inflammatory disease and type of bacteria in the outcome for treatment failure

**Bold values represent statistical significance (*p* < 0.05)

## Results

Data were available from 57 patients subjected to DAIR and exchange of modular components for acute hip (31/57) or knee (26/57) periprosthetic infection: 37 in the multidrug-sensitive bacteria group, 11 in the methicillin-resistant *Staphylococcus aureus* group and 9 in the Gram negative multidrug-resistant bacteria group. The characteristics of the groups are summarized in Table 3. There was no statistically significant difference in the distribution of patient’s risk factors for PJI between the groups. All patients were submitted to DAIR between 10 and 21 days after the index surgery. There was no statistical difference in outcome regarding the time between the index surgery and DAIR procedure (*p* = 0,108). The median follow-up time was 5.0 (range 3.0–7.0) years. The multidrug-sensitive bacteria group (37 patients) contained 70% of Gram positive (26/37), which was composed most frequently by *Staphylococcus aureus* (19/26) followed by *Staphylococcus epidermides* (7/26). Gram negative multidrug-sensitive bacteria (11/37) was composed by *Escherichia coli* (4/11), Acinetobacter (2/11), *Enterobacter cloacae* (2/11) and others. There was no case of polymicrobial infection diagnosed during the first DAIR procedure.

**Table 3:** Demographic data

	Multidrug-sensitive	*Methicillin-resistant Staphylococcus aureus*	Other multidrug- resistant bacteria	P
Number of patients	37	11	9	–
TKA	18 (50.0%)	4 (36.4%)	3 (33.3%)	0.55
THA	18 (50.0%)	7 (63.6%)	6 (66.7%)	0.55
Age - median (interquartile range)	68 (10)	68 (9)	65 (5)	0,99
Follow-up time - median Interquartile range)	5 (2)	5 (2)	5 (2)	0.68
Time of infection after initial surgery (interquartile range)	25 (20)	25 (16)	21 (18)	0.68
Male gender	15 (41.7%)	5 (45.5%)	2 (22.2%)	0.51
Osteoarthritis	33 (91.7%)	11 (100%)	9 (100%)	0.42
Diabetes	8 (22.2%)	5 (45.5%)	1 (11.1%)	1.00
Smoking	1 (2.8%)	0	0	0.21
Rheumatic disease (Rheumatoid Arthritis)	3 (8.3%)	3 (27.3%)	2 (22.2%)	0.16

It was not necessary to adjust initial antibiotic therapy (teicoplamin and amikacin) based in the results of intra operative cultures in the multidrug-sensitive bacteria and MRSA groups. In the multidrug-resistant bacteria group, it was necessary to adjust the antibiotic in 6/9 (66%) cases regarding bacteria resistance. Of these 6 patients, 3 had failure treatment as outcome (2 *Klebsiella pneumoniae* and 1 *Escherichia coli*) which had to be changed accordingly (meropenem and tigecycline, respective).

Therapeutic success after DAIR with the exchange of modular components was different between groups: treatment failure was 8.3% in the multidrug-sensitive bacteria group, 18.2% in the methicillin-resistant *Staphylococcus aureus* group and 55.6% in the Gram negative multidrug-resistant bacteria group (*p* = 0.005) (Table 4).

**Table 4:** Treatment failure between groups

	Multidrug- sensitive	Methicillin-resistant Staphylococcus aureus	Other multidrug- resistant bacteria	Total	P
Failure	3 (8.30%)	2 (18.20%)	5 (55.6%)	10 (18%)	0.005

Of the 10 cases that failed initial treatment, 5 had a successful second DAIR procedure without the need for revision or replacement of metal implants (4 by multidrug-resistant bacteria and 1 multidrug-sensitive), 3 underwent two-stage revision with the placement of a cement spacer between surgeries (one by MRSA, one by multidrug-resistant *Escherichia coli* and the other by methicillin-sensitive *Staphylococcus aureus*), and 2 cases of TKA progressed to transfemoral amputation due to infection after a failed two-stage revision. About the two patients that evolved to amputation, one had an infection by MRSA and the other initially had an infection by a multidrug-sensitive *Escherichia coli*, which evolved to a polymicrobial infection after many attempts of debridement. None of these cases presented new recurrences of the infection until our last control.

The significant variables of the model were rheumatic disease (*p* = 0.04), infection by multidrug-sensitive bacteria (*p* = 0.034), infection by methicillin-resistant *Staphylococcus aureus* (*p* = 0.01) and infection by multidrug-resistant Gram-negative bacteria (*p* = 0.048). The odds ratio for infection failure due to rheumatic disease was 45.1, related to methicillin-resistant *Staphylococcus aureus* was 58.5 and related to multidrug-resistant gram-negative bacteria was 49.9.

## Discussion

Our study demonstrated that the general incidence of infection recurrence in the treatment with DAIR was 18%. Controlling for other variables, only two risk factors among all evaluated were significant: multidrug-resistant bacterial infection (MRSA or Gram negative) and patients with previous rheumatic diseases.

The treatment of periprosthetic hip and knee infections is a challenge for both the orthopedist and the patient and is one of the most feared complications after arthroplasty [2]. In acute infections, the main objective is to avoid biofilm formation around the prosthetic material through a careful procedure that includes irrigation, debridement, the exchange of modular components and broad-spectrum antibiotic therapy [8]. The prognosis in these cases can be discouraging when associated with risk factors such as multidrug-resistant bacteria, rheumatic diseases, diabetes and smoking [2, 9, 10, 12, 14, 15].

The recurrence of infection after the initial treatment of a periprosthetic joint infection with DAIR varies in the literature between 14 and 69% [10, 16, 17]. The highest number of cases study, as described by Cochran et al. [10], presented a reinfection rate of 28,2% in the first year and 43,2% after 6 years of the surgical procedure with DAIR. The reinfection rate of 18% (10/57) found in our study agrees with the values described in the literature and is acceptable for this type of treatment.

Regarding the type of microorganism causing the infection, our study showed a treatment failure rate of only 8.3% (3/37) when the bacterium found in the culture was multidrug-sensitive. In the MRSA group, we observed a failure rate of 18.2% (2/11). The odds ratio for recurrence of infection in the MRSA group, in comparison with the other groups, was 58.5 (*p* = 0.01). More outstanding, the group of patients with Gram negative multidrug-resistant bacteria had a failure rate of 55.6% (5/9), with an odds ratio of 49.9 (*p* = 0.048). Most studies in the literature corroborate our findings pointing to multidrug-resistant bacteria as a factor of poor prognosis in the success of PJI treatment [18, 19]. The reasons for worst outcomes in resistant organism are multifactorial, such as limited choose of effective antibiotics, more aggressive biological behavior related to biofilm production and greater virulence [2, 20]. Bradbury et al. [18] found an infection recurrence rate of 84% after irrigation and debridement treatment when the bacteria found in the cultures was MRSA compared to the overall failure rate of 18%. Based on these findings, some authors have gone so far as to recommend a two-stage revision in acute infection caused by MRSA [18, 19]. In contrast, our findings demonstrate a reasonable chance of success in MRSA compared to multidrug-resistant Gram-negative bacteria, which raise a question regarding the role DAIR in these patients. Hsieh et al. [21] compared the infection recurrence rate after irrigation and debridement of Gram-positive bacteria with Gram-negative bacteria and obtained a success rate of 47% vs. 27%, respectively. Even when compared to other types of initial surgical procedures, non-MRSA multidrug-resistant bacteria tend to have worse outcomes than MRSA. Vaso et al. showed a failure rate of 33% for non-MRSA multidrug-resistant bacteria versus 10% for MRSA in two-stage revision knee arthroplasty. However, some authors did not find a significant difference in results when comparing multidrug-sensitive and multidrug-resistant bacteria [22, 23]. Most studies, however, have used small sample sizes, which hinders further conclusions.

Regarding antibiotic therapy management, in only 6/57 cases (all multidrug resistance bacteria) the initial antibiotic protocol was not suitable based on bacteria resistance profile. Half of those cases (3/6) had failed DAIR procedures. We analyze the risk and benefit of expanding the spectrum of initial antibiotic therapy protocol for PJI, but due to the concern in increasing bacteria resistance and the fact that nowadays all patients with suspected PJI are submitted to joint aspiration previously to any surgical procedure (with very few incidence of negative cultures and dry aspiration), we find it unsuitable to modify our existent protocol.

Another risk factor that was statistically significant in our study was the presence of associated rheumatic disease with an odds ratio for treatment failure of 45.1. It is already well established in the literature that rheumatic diseases is an independent risk factor for PJI [24]. Bongartz et al. [25] demonstrated an increased risk of PJI in patients with rheumatic disease, with an odds ratio of 4.08 (95% CI 1.35–12.33).

The present study has limitations regarding its retrospective design and sample size. Despite the increase in bacterial drug profile resistance in the last decade, the low incidence of PJI, particularly related to multidrug-resistant organism, results in the absence of large studies involving these types of microorganisms in medical literature.

The prevention and treatment of acute prosthetic infection is extremely important today because of its severity, difficulty to treat, and its large contribution to the total number of failed joint replacements. As the number of joint replacements is expected to rise, this problem will only become more relevant in the next years. It is important to identify multi-drug resistant bacteria and inflammatory disease as factors of poor prognosis in the treatment of acute infection with DAIR. These data may warrant future clinical trials comparing DAIR and revision arthroplasty in this high-risk situation, and at present allow for a more informed treatment decision of surgeons, infection specialists and patients.

## Conclusion

Infection by MRB, MRSA and inflammatory arthritis were independent risk factors for failure after treatment with DAIR for acute hip and knee periprosthetic infections. In the presence of these organisms, DAIR should be consider with caution.

## Data Availability

The datasets generated and/or analyzed during the current study are not publicly available because it belongs to the personal data of our institution and are used in other articles related to this topic but are available from the corresponding author on reasonable request.

## Abbreviations

DAIR: Debridement, antibiotics and implant retention
PJI: Periprosthetic joint infection
THA: Total hip arthroplasty
TKA: Total knee arthroplasty
MRSA: Methicillin-resistant *Staphylococcus aureus*
VRE: Vancomycin-resistant *Enterococcus spp*
MSIS: Musculoskeletal Infection Society
